# Vocal sequences suppress spiking in the bat auditory cortex while evoking concomitant steady-state local field potentials

**DOI:** 10.1038/srep39226

**Published:** 2016-12-15

**Authors:** Julio C. Hechavarría, M. Jerome Beetz, Silvio Macias, Manfred Kössl

**Affiliations:** 1 Institut für Zellbiologie und Neurowissenschaft, Goethe-Universität, Frankfurt/M., Germany; 2 Department of Psychological & Brain Sciences, Johns Hopkins University, Baltimore, USA

## Abstract

The mechanisms by which the mammalian brain copes with information from natural vocalization streams remain poorly understood. This article shows that in highly vocal animals, such as the bat species *Carollia perspicillata,* the spike activity of auditory cortex neurons does not track the temporal information flow enclosed in fast time-varying vocalization streams emitted by conspecifics. For example, leading syllables of so-called distress sequences (produced by bats subjected to duress) suppress cortical spiking to lagging syllables. Local fields potentials (LFPs) recorded simultaneously to cortical spiking evoked by distress sequences carry multiplexed information, with response suppression occurring in low frequency LFPs (i.e. 2–15 Hz) and steady-state LFPs occurring at frequencies that match the rate of energy fluctuations in the incoming sound streams (i.e. >50 Hz). Such steady-state LFPs could reflect underlying synaptic activity that does not necessarily lead to cortical spiking in response to natural fast time-varying vocal sequences.

Animal vocalizations play a role as behavioural transmitters (“ethotransmitters”) because they can act on other organisms triggering energetically costly responses through elevated neural activity and activation of the somatic, autonomic and endocrine systems^1^,^2^. Vocalizations produced in alarmful situations are perhaps one of the best examples of acoustic signals as ethotransmitters. When under duress or when in the presence of danger, most vertebrate species produce sounds that (among other functions) serve to advertise the ongoing discomfort situation. Calls uttered in distress circumstances are known as “distress calls”, “alarm calls”, or “screams”^2^,^3^,^4^,^5^,^6^,^7^,^8^,^9^,^10^,^11^, and they have profound effects in the listeners’ physiology and behaviour, such as boosting gene expression, elevating heart-rate, activating the neuroendocrine axis, and evoking exploratory and mobbing behaviours in conspecifics^5^,^6^,^7^,^9^,^10^,^12^,^13^,^14^,^15^,^16^,^17^,^18^,^19^,^20^,^21^,^22^.

The physical attributes of distress vocalizations have been intensively studied (see^3^,^4^,^6^,^16^,^19^,^23^,^24^,^25^,^26^,^27^,^28^,^29^) but, at present, comparably little is known about how distress signals are processed in the listeners’ brain, especially at the level of the auditory cortex (AC). Distress vocalizations are produced in the form of “sequences” composed of individual sound units defined as “syllables”^4^,^19^,^24^,^25^,^30^,^31^. In the AC, representing the information carried by single syllables embedded within a vocal sequence can be affected by phenomena such as forward suppression and adaptation. These physiological phenomena cause a reduction of spike activity over time^32^,^33^,^34^,^35^,^36^,^37^ and they could potentially affect the cortical representation of distress information.

The main aim of this manuscript is to investigate how the AC responds to distress sequences uttered by conspecifics. We recorded spikes and local field potentials (LFPs) evoked by distress sequences in the AC of the bat species *Carollia perspicillata*. LFP and spike activity can be measured simultaneously by analysing different frequency bands of the voltage waves recorded using extracellular electrodes, i.e. <200 Hz (LFP) and between 300–3000 Hz (spiking). Spike signals refer to action potentials generated by cortical neurons, while LFPs result from the summation of synaptic activity in the cortex, activity in extra-cortical areas and the slow components of spikes produced by neurons near the recording electrodes^38^,^39^,^40^.

Our goal was to assess whether forward suppression decreases cortical spiking in response to lagging portions of the distress sequences (as it could be predicted from the currently available literature on forward suppression^34^,^35^,^41^,^42^), and whether this reduction in spike activity could prevent LFPs from being “entrained” by the distress streams, to create so-called local steady-state responses (LSSRs). LSSRs have been widely used for studying auditory function in the healthy and diseased brain^43^,^44^,^45^,^46^,^47^,^48^,^49^, and they have been interpreted as an indication of the neural tissue being capable to temporally track sensory information flows^45^,^47^,^50^,^51^. Yet, at present, the neural generators that contribute to LSSRs remain poorly understood.

*C. perspicillata -*the bat species used as animal model for this study*-* is known to produce two basic types of distress sequences (see^4^): short sequences containing a single group of syllables (Fig. 1a); and long sequences containing multiple syllable groups defined as multi-syllabic bouts (see Fig. 1b–c). Distress sequences uttered by *C. perspicillata* are composed of syllables repeated at rates of ~14 ms^4^. This repetition rate is faster than that observed while *C. perspicillata* is calling in other scenarios, such as echolocation, in which the shortest calling intervals are above 26 ms^42^,^52^,^53^.

We observed that -as predicted- forward suppression participates in the processing of distress sequences. Even if cortical spiking undergoes suppression, simultaneously recorded LFPs show a steady-state response that could reflect underlying cortical synaptic activity resulting from extra-cortical inputs to the auditory cortex.

## Results

**Figure 1:**
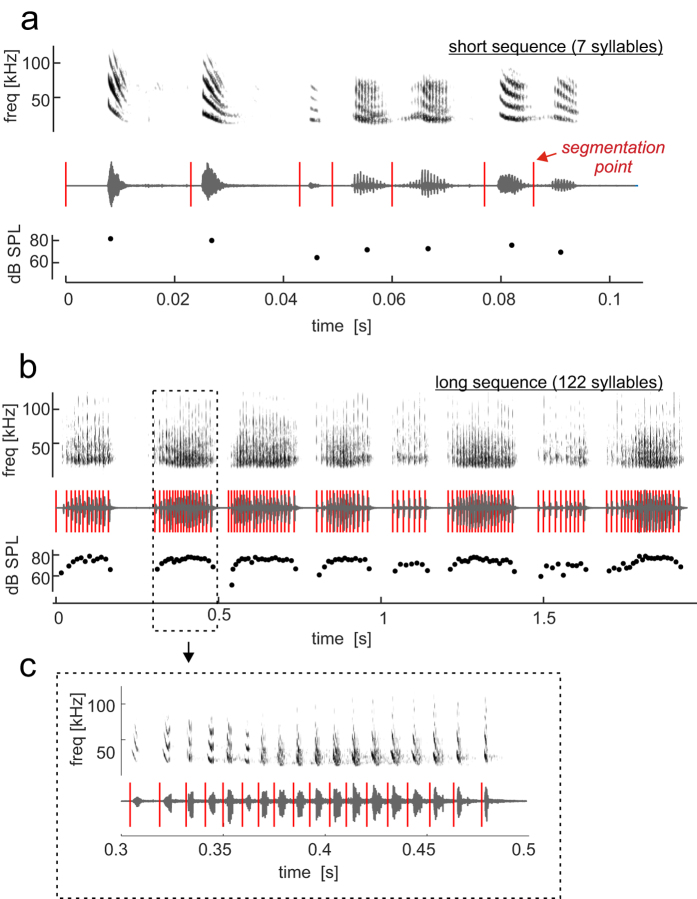
The two distress sequences used as stimuli to study the activity of cortical neurons. (**a**) Short sequence. (**b**) Long sequence containing eight multi-syllabic bouts (i.e. groups of syllables). For each sequence, the spectrogram, oscillogram and amplitude (per syllable and in dB SPL) is shown from top to bottom. Amplitude was calculated as the root mean square of each syllable, and transformed into dB SPL by comparing against a reference tone (see Methods). Vertical red lines on top of the oscillograms indicate the time points at which each sequence was cut for creating several sequence “segments”, each of which contained only one syllable. Segments were used for calculating the neurons’ expected responses (see also Figs 2 and 4, and Methods). (**c**) Shows a zoom-in on the second multisyllabic bout of the long distress sequence.

Neuronal responses were studied in both cortical hemispheres of animals that were lightly anesthetized (see Methods). Two distress sequences of different lengths were used as stimuli (Fig. 1). These two sequences were chosen because they represent typical examples of a short distress sequence (Fig. 1a) and a long sequence containing multisyllabic bouts (Fig. 1b, see methods and^4^ for a description of distress sequences produced by *C. perspicillata*). Responses were studied by playing segments of the two sequences, as well as the sequences in their natural form, that is, with their temporal structure exactly as they were broadcasted by the bats. Segments and sequences were played in a pseudo-randomized order at intervals of 400 ms. This stimulation procedure aims to assess the effect of temporal patterning on cortical processing of single syllables, by comparing responses to syllables presented in isolation with responses to the same syllables when embedded in a sequence. Recordings were made from the tonotopic fields of the auditory cortex (that is: AI, AII and AAF, see^54^), although we cannot discard that some measurements could have been from high frequency non-tonotopic areas (so-called HF fields^54^). That most of the recordings were performed in the tonotopic cortical fields was determined by inspecting the frequency receptive fields of neurons recorded across cortical locations.

### Cortical spiking in response to short distress sequences

Cortical spiking in response to the short distress sequence was studied in 116 and 119 neurons from the left and right AC, respectively. The segmentation of the short sequence is illustrated in Fig. 1. Each segment contained only one syllable and the temporal structure of the short sequence could be obtained by concatenating its segments in the proper order. We wanted to compare neuronal responses to the natural sequences (defined as “observed responses” from here on) with “expected responses” that were estimated from the spike activity evoked by the different sequence-segments. Expected responses resulted from realigning the spike-times obtained in response to the sequence-segments based on the segments’ positions within the natural sequence.

**Figure 2:**
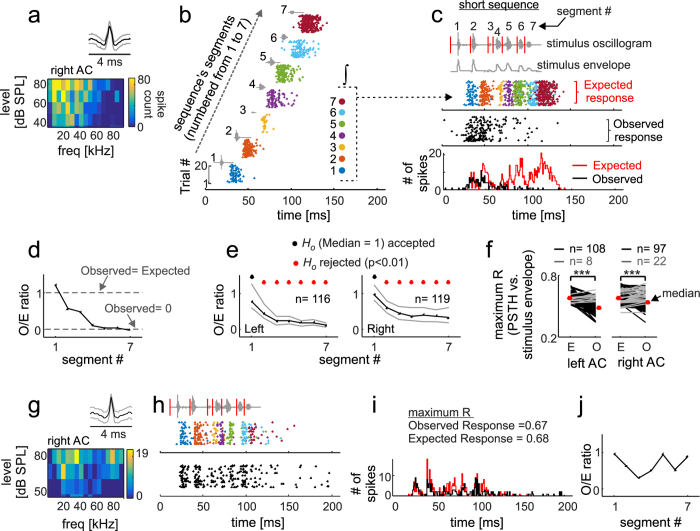
Auditory cortex (AC) responses to a “short” distress sequence. (**a**) Spike waveform (median in black, 25^th^ and 75^th^ percentiles in grey) and frequency-level receptive field of a cortical neuron. (**b**) Response of the same neuron to 20 trials of each of the segments of the short sequence. Segments have been numbered from 1 to 7 according to the temporal order in which they occurred in the short sequence. Dots indicate spike-times (color-coded by segment) aligned based on the segments’ position in the non-segmented sequence. (**c**) Shows (from top to bottom): the oscillogram and energy envelope of the short distress sequence; the raster plot of the “expected response” (colored dots) to the short sequence for the same neuron represented in (**b**); the raster plot obtained when playing the non-segmented short sequence (“observed response”, black dots); and the observed and expected post-stimulus time histograms (PSTHs, 1 ms bin-size). Note that the expected and observed responses calculated for this neuron differed towards the end of the sequence. (**d**) The observed-to-expected spike count ratio (*O/E ratio*) calculated for each segment of the short sequence. (**e**) Median *O/E ratios* (black line, 25^th^ and 75 percentiles are represented in grey) calculated from all the neurons studied in each cortical hemisphere. The results of a Sign Test, that compared the median *O/E ratio* distributions against “1”, are given. (**f**) Maximum cross-correlation coefficient (maximum R) obtained between the stimulus envelope and the expected and observed PSTHs. For this procedure, the envelope was down-sampled to a 1-ms resolution. Black lines indicate neurons in which the expected PSTH was better cross-correlated with the sequence envelope than the observed PSTH. Grey lines indicate the opposite trend. Red dots indicate median population values. The results of paired Wilcoxon Signed-Rank tests are given (***p < 0.001). (**g**–**j**) Show the spike waveform and frequency-level receptive field (**g**), the expected and observed raster plot (**h**), the expected and observed PSTHs (**i**), and *O/E ratio* curve (**j**), of one neuron whose expected response was essentially similar to its observed response.

Figure 2a–b illustrates the procedure used for obtaining the expected response in one neuron. This neuron fired strongly to each of the seven segments of the short sequence (Fig. 2b). Consequently, its expected response (Fig. 2c) suggested that the neuron could potentially track the short sequence until its very end. The latter can be seen in the raster plot that reunites the spike-times to all 20 trials of each segment of the short sequence (Fig. 2c, coloured raster), and in the expected post-stimulus time histogram (expected PSTH,) obtained by counting the spikes in the expected raster plot over 1-ms bins (Fig. 2c, bottom panel).

A comparison between the expected and observed responses for the example neuron represented in Fig. 2a–d revealed a suppression in response to lagging syllables of the short distress sequence. For example, spikes that were expected to occur ~70 ms after the start of the sequence simply were not there (Fig. 2c, middle and bottom panels; see also Supplementary Figure S1 for further examples of suppression driven by the short distress sequence). To characterize the suppression observed when comparing observed and expected responses, observed-to-expected spike-count ratios (*O/E ratios*) were calculated. *O/E ratios* were obtained by dividing the number of spikes occurring within the time borders of each segment in the observed and expected responses. The *O/E ratio* curve obtained for the example neuron represented in Fig. 2a–d showed values close to “1” for the leading sequence-segment, and values close to “0” for lagging segments (see Fig. 2d). *O/E ratios* close to “1” indicate that the observed and expected responses are similar to each other, while values close to “0” indicate a lack of spike activity in the observed response. Note that segment-dependent *O/E ratios* provide an idea on how suppression evolves over entire sequences, but one should be aware that spikes occurring within the time borders of a specific segment might not be necessarily driven by the syllable in that segment.

Median *O/E ratios* were calculated for each cortical hemisphere by pooling data from 116 and 119 units recorded in the left and right AC, respectively (Fig. 2e). The calculated median *O/E ratio* curves showed the same trend described in the preceding text, that is, *O/E ratios* close to “1” for leading segments and *O/E ratios* close to “0” for lagging segments of the short sequence. The latter suggests that response suppression occurs in most cortical neurons of both hemispheres when a bat hears a short distress sequence. The statistical significance of this suppression was assessed using a Wilcoxon signed-rank test, whose null hypothesis was that the distribution of *O/E ratios* calculated for each segment had a median that was equal to “1”. For both cortical hemispheres, the null hypothesis of the test was accepted (*p *> 0.05) only for *O/E ratios* corresponding to the first segment. In other words, at the population level, only during the first segment of the short sequence could the “observed response” be predicted based on the “expected response”.

**Figure 3:**
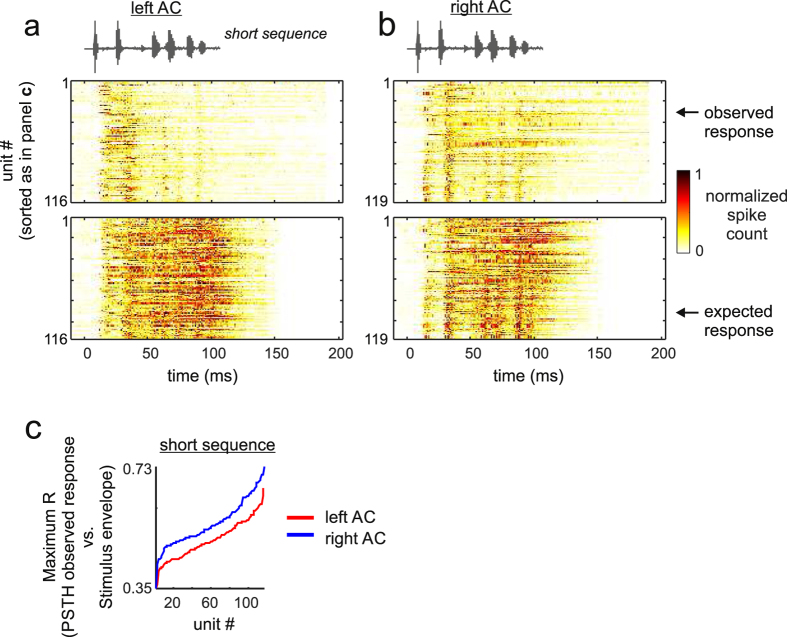
Population raster plots of the observed and expected responses obtained in response to the short distress sequence in the left (**a**) and right (**b**) hemispheres of the auditory cortex. Population responses are shown as colormaps, in which the colors indicate the normalized spike-counts of the units studied. In each unit, spike-count was normalized to the maximum number of spikes obtained in time-bins of the unit’s observed and expected PSTHs (see also Fig. 2). Units are ordered according to their “Maximum R” obtained by cross-correlating the observed post-stimulus time histogram of a particular unit with the energy envelope of the vocal sequence that evoked the response (i.e. the short sequence). In other words, units whose response is represented at the bottom of the colormaps were classified as “better followers” of the sequence envelope than units that appear at the top. The “Maximum R” values obtained in each unit in response to the short sequence are shown in (**c**).

To further investigate whether expected and observed responses were different from each other, we calculated cross-correlation coefficients between the energy envelope of the short distress sequence (see sequence envelope in the upper panel of Fig. 2c), and the expected and observed PSTHs. For this procedure, the sequence envelope was down-sampled to a temporal resolution similar to that in the PSTHs (i.e. 1 ms). This analysis aims to assess if the “observed response” of some neurons carries information that correlates with the temporal energy flow of the distress sequence used as stimulus, something that could occur even in situations in which the observed response is suppressed when compared to the expected response. The results revealed that in most neurons of both cortical hemispheres, expected responses were better correlated with the stimulus envelope than were observed responses (Fig. 2f). This effect was significant at the population level in both hemispheres (paired Wilcoxon signed-rank test, *p* = 4*10^−19^ (left AC), *p* = 1*10^−12^ (right AC)). However, we did notice that the response of a few neurons (grey lines in Fig. 2f) did not show the typical decrease in the PSTHs vs. stimulus-envelope cross-correlation coefficient. These were neurons that managed to fire throughout the short distress sequence, even if their response appeared as suppressed at certain PSTH time bins. An example of one of these neurons is shown in Fig. 2g–j (see also the example neuron in Supplementary Figure S2a-b). Although these “atypical” neurons were minority in each hemisphere, in response to the short sequence, they were more likely to be encountered in the right AC, where they represented the 18.5% (22/119) of the studied neurons, while they represented only 7% (8/116) of the neurons studied in the left AC. The inter-hemispheric differences in terms of suppression in response to the short-distress sequence were also apparent in population raster plots (Fig. 3). Observed and expected population raster plots were more similar to each other in the right (Fig. 3b) than in the left hemisphere (Fig. 3a).

### Cortical spiking in response to long distress sequences

Most of the distress sequences broadcasted by bats in natural scenarios contain more than 10 syllables and several multi-syllabic bouts^4^. Therefore, to better understand how the bat cortex copes with the temporal patterning of information that is encountered more often in natural scenarios, we studied neuronal responses to a long distress sequence containing eight multi-syllabic bouts and a total of 122 syllables (see sequence in Fig. 1b–c).

**Figure 4:**
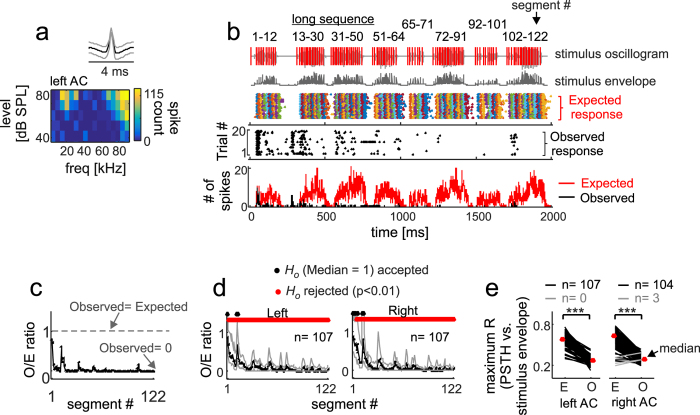
Auditory cortex responses to a “long” distress sequence. (**a**) Spike waveform (median in black, 25^th^ and 75^th^ percentiles in grey) and frequency-level receptive field of a cortical neuron. (**b**) From top to bottom: the oscillogram and energy envelope of the long distress sequence (segment numbers are indicated on top); the raster plot of the “expected response” (colored dots) to the long sequence for the same neuron represented in **a**; the raster plot obtained when playing the non-segmented long sequence (“observed response”, black dots); and the observed and expected post-stimulus time histograms (PSTHs, 1 ms bin-size). Note that spiking was strongly reduced in the observed response when compared to the expected response. (**c**) The observed-to-expected spike count ratio (*O/E ratio*) calculated for each segment of the long sequence. (**d**) Median *O/E ratios* (black line, 25^th^ and 75 percentiles are represented in grey) calculated from all the neurons studied in each cortical hemisphere. The results of a Sign Test, that compared each median *O/E ratio* distribution against “1”, are given. (**e**) Maximum cross-correlation coefficient (maximum R) obtained between the sequence’s envelope and the expected and observed PSTHs. Black lines indicate neurons in which the expected PSTH was better cross-correlated with the sequence’s envelope than the observed PSTH. Grey lines indicate the opposite trend. Red dots indicate median population values. The results of paired Wilcoxon Signed Rank tests are given (***p < 0.001).

The procedure followed for studying responses to the long sequence was similar to that described in the preceding text for the short sequence. Figure 4 shows the response of an example neuron to the long sequence. The expected response (Fig. 4b, coloured raster) predicted that this neuron could track the entire sequence until its very end. However, the observed response (Fig. 4b, black raster) showed that spiking decreased as the sequence evolved, with an almost total lack of spiking in response to segments 65–101 (Fig. 4b). This reduction in spiking over time rendered *O/E ratios* that were close to 0 for lagging segments (Fig. 4c). Further examples of suppression in response to the long distress sequence can be found in Supplementary Figure S3.

**Figure 5:**
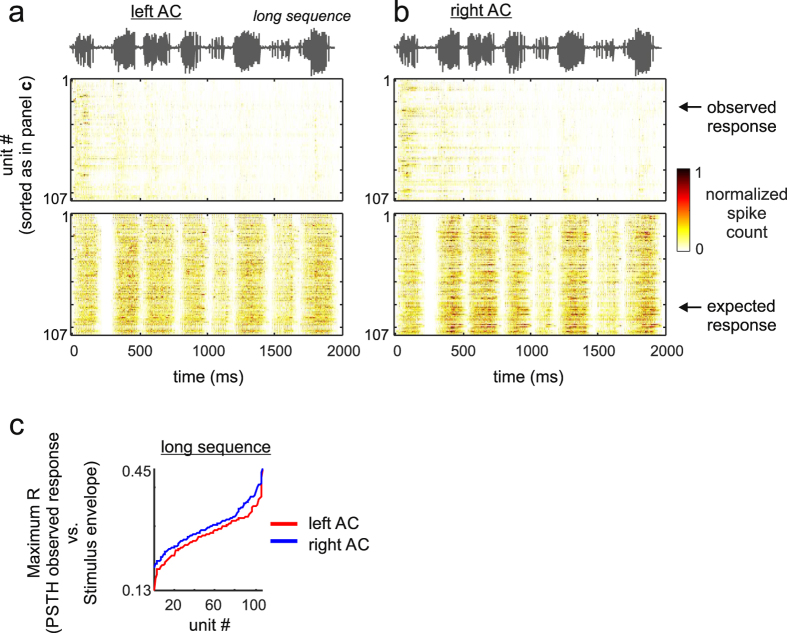
Population raster plots of the observed and expected responses obtained in response to the long distress sequence in the left (**a**) and right (**b**) hemispheres of the auditory cortex. Population responses are shown as colormaps, in which the colors indicate the normalized spike-counts of the units studied. In each unit, spike-count was normalized to the maximum number of spikes obtained in time-bins of the unit’s observed and expected PSTHs (see also Fig. 4). Units were ordered according to their “Maximum R” obtained by cross-correlating the observed post-stimulus time histogram of a particular unit with the energy envelope of the natural sequence that evoked the response. In other words, units whose response is represented at the bottom of the colormaps were classified as “better followers” of the sequence envelope than units that appear at the top. The “Maximum R” values obtained in each unit in response to the long sequences are shown in (**c**).

At the population level, the median *O/E ratio* curves calculated from 107 neurons in each hemisphere, also revealed the presence of strong suppressive effects that appeared to dominate cortical responses to the long distress sequence. For example, in both hemispheres, only the leading segments (i.e., before segment #15) rendered *O/E ratio* distributions whose median was not significantly different from “1” (Fig. 4d). The maximum cross-correlation coefficient calculated between the envelope of the long sequence and the observed and expected PSTHs revealed that, in each hemisphere, the neurons’ capability to track the sequence envelope was rather poor. To illustrate, the percentage of neurons that were equally good in tracking the long sequence according to their observed and expected PSTHs was very low in both hemispheres (Fig. 4f, grey lines), i.e. 0% in the left AC and 2.8% (3/107 neurons) in the right AC. That there were no apparent inter-hemispheric differences in terms of suppression in response to the long distress sequence was also observed in population raster plots (Fig. 5).

The lack of inter-hemispheric differences in terms of suppression driven by long sequences contrasts with data obtained in response to the short sequence, for which 18.5% (22/119) of the neurons studied in the right AC had a comparable sequence-tracking ability in their expected and observed PSTHs (Fig. 2f, right panel, see also population rasters in Fig. 3). Note that even neurons that were classified as “sequence followers” in response to the short sequence were incapable of tracking the information contained in the long sequence (see Supplementary Figure S2 for an example response to the long sequence in a neuron that could track the short sequence).

### Suppressed spiking and concomitant steady-state LFPs

The finding that the spike activity of most cortical neurons does not keep up with the temporal flow of information is consistent with results from experiments conducted in other vertebrate species^34^,^41^,^55^,^56^. Of course, that suppression occurs at the cortical level does not necessarily imply that distress information is not being processed in auditory stations outside of the AC. To test this idea, we studied cortical local field potentials recorded simultaneously to the spike activity. LFPs are slow signals (slower than spike activity, i.e. <200 Hz (LFP) vs. 300–3000 Hz (spikes)) and the general consensus is that LFP waves originate from a mixture of synaptic activity at the boundaries of the recording electrode, and from activity in distant extra-cortical areas that is strong enough for reaching the cortical electrodes^38^. We reasoned that LFP signals containing voltage fluctuations that follow the energy envelope of a given acoustic sequence would indicate that extra-cortical auditory stations can keep up with the sequences’ temporal information flow, even if the AC is not. Note that cortical LFPs will ultimately depend upon subcortical processing, regardless of whether the recorded LFPs originate only from local synaptic activity created by feed-forward inputs to the cortex, or whether they also contain information from electric fields generated in extra-cortical structures. For studying LFPs, we focussed on signals evoked by the long distress sequence because only that sequence was long enough (i.e. ~2 s) for triggering several cycles of slow LFP activity (i.e. activity <15 Hz).

**Figure 6:**
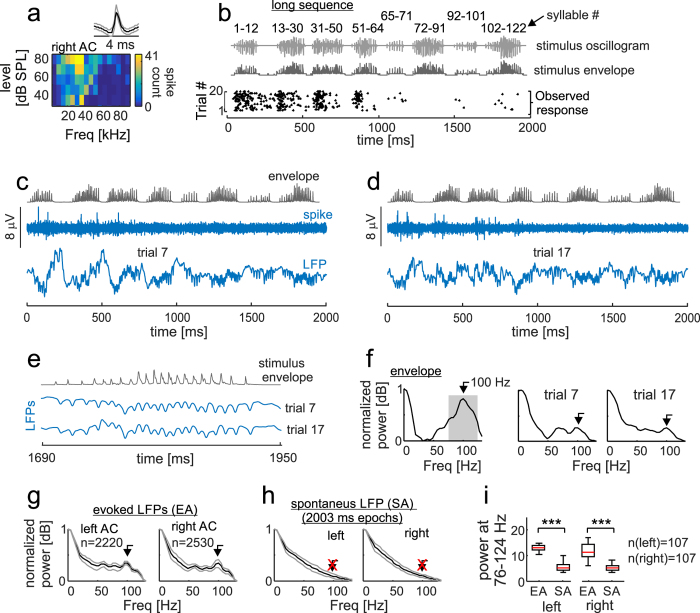
Steady-state local field potentials obtained in response to a long distress sequence. (**a**) Shows the spike waveform (median in black, 25^th^ and 75^th^ percentiles in grey) and frequency-level receptive field of one neuron recorded in the right auditory cortex. (**b**) From top to bottom: oscillogram and energy envelope of the long distress sequence (syllable numbers are indicated), and the raster plot obtained when playing the sequence (“observed response”, black dots). (**c**–**d**) have a similar structure and show spike and local field potentials (LFPs) obtained in the same neuron represented in (**a**–**b**) during two trials of the long sequence. **(c–d**) Show (from top to bottom): the energy envelope of the distress sequence; the spike activity; and the LFP waves. (**e**) Shows a zoom-in on the sequence envelope and on the LFPs obtained in the same two trials represented in (**c**–**d)** (i.e. 1690–1970 ms after sequence start). (**f**) Shows the spectrum of the sequence envelope and the spectra of the LFP trials represented in (**c**–**d**). Spectra are represented in a logarithmic scale (i.e. in dBs) and are normalized to their maximum value. Note that the spectrum of the sequence-envelope peaked at 100 Hz, and a similar peak was found in the spectra of LFP trials. (**g**) Median normalized spectra obtained from all trials studied in each cortical hemisphere. Grey lines represent 25^th^ and 75^th^ percentiles. (**h**) Median spectra of LFP epochs recorded in the absence of acoustic stimulation (spontaneous activity, SA). SA epochs had a duration similar to that used for studying epochs of evoked activity (EA). No peak at 100 Hz was found in the median SA spectrum. (**i**) Comparison of median LFP power obtained in the 76–124 Hz band (see shaded area in **f**). Median power was calculated for each recording site by averaging across epochs obtained with or without acoustic stimulation (EA and SA, respectively). In the box-plots, central marks are medians, boxes indicate 25^th^ and 75^th^ percentiles, and the whiskers extend to the most extreme data points not considered outliers. The results of a paired Wilcoxon Signed-Rank tests are given (***p < 0.001).

Two example LFP signals recorded in one neuron during two trials of the long sequence are shown in Fig. 6c–d, together with the spike trace recorded simultaneously to the LFP signals. Note that this neuron fired to the beginning of the first four multi-syllabic bouts, but its spike activity was strongly reduced after 1000 ms into the sequence (Fig. 6b). In the two trials represented in Fig. 6c–d, the start of the sequence triggered a slow oscillation in the LFP. Riding on this slow oscillation, there were faster small-amplitude energy fluctuations that were present even in response to the last multi-syllabic bout (Fig. 6e), a time window during which no spikes occurred. Note that the temporal pattern of LFP activity observed in response to the last multi-syllabic bout appeared to follow the energy flow observed in the corresponding portion of the sequence’s envelope (Fig. 6e).

The correspondence between the sequence’s temporal structure and that of the LFPs was studied by calculating spectra of both the sequence’s energy envelope and single trial LFPs (see Methods). In the spectrum of the long sequence’s energy envelope, there was a peak at 100 Hz (Fig. 6f). A similar peak (at ~100 Hz) was found in the spectra of single trial LFPs (Fig. 6f), and in the median spectra of all trials recorded in both hemispheres (Fig. 6g, trials studied in 107 units from each hemisphere). Similar results were obtained from LFPs evoked by the short sequence (see Supplementary Figure S4 for data on the short sequence). Altogether, these data indicate that listening to a distress sequence triggers a *local steady-state response* (LSSR) in the cortex. Such LSSR is measurable in cortical LFPs, even if cortical neurons fail to spike in response to syllables that occur towards the end of the sequences.

The observed LSSR was not apparent in randomly chosen epochs of spontaneous activity that had the same length of the epochs used to study evoked activity (Fig. 6h). The latter was statistically confirmed by comparing the median power (in the band from 76–124 Hz) measured in evoked and spontaneous LFPs obtained in each studied recording site (Fig. 6i, paired Wilcoxon signed-rank test, *p *< 10^−30^ for both cortical hemispheres). Power was measured in the frequency band from 76–124 Hz, because this band carried the strongest energy fluctuations in the spectrum of the long-sequence’s energy envelope (see shaded area in Fig. 6f).

### Suppression and steady-state occur in different frequency bands of the LFP

LFPs measured in the AC are formed by a blend of low frequency and high frequency waves (for examples of raw LFP traces see Fig. 6c and d, and Supplementary Figure S4). We wanted to study whether the response suppression observed in the spike activity of cortical neurons was apparent in either slow or fast LFPs. As in the analysis of LFPs described in the preceding section, for this analysis we focussed on LFPs obtained in response to the long sequence only. The slow LFP component (sLFP) was obtained by re-filtering LFPs between 2–15 Hz (corresponding to the alpha-theta band in the EEG). On the other hand, fast LFPs (fLFPs) were obtained by filtering between 76–124 Hz, the frequency range that was best represented in the spectrum of the envelope of the long distress sequence (see shaded area in Fig. 6f).

**Figure 7:**
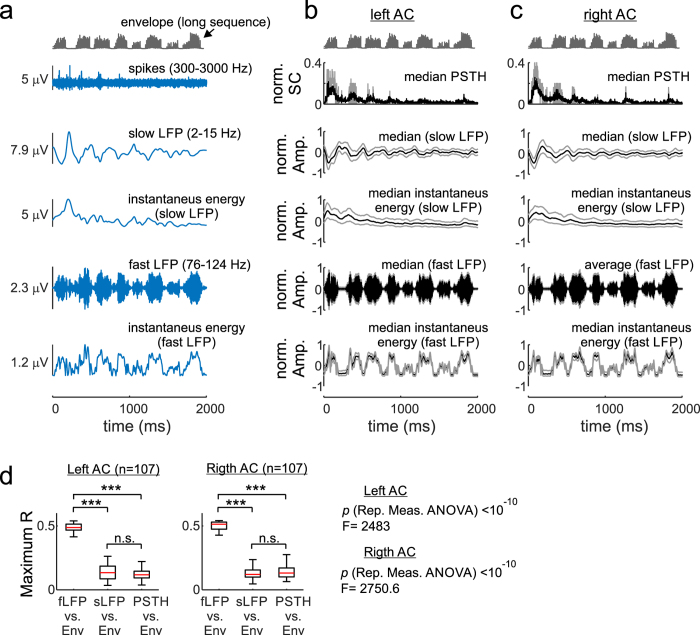
Relationship between stimulus envelope, spiking, and slow and fast LFPs (sLFPs and fLFPs, respectively). (**a**) Shows LFP and spike data recorded during one trial of the long distress sequence. The data corresponds to the same neuron represented in Fig. 6a–f. (**a**) Shows (from top to bottom): the envelope of the long distress sequence; the spike trace obtained in one trial of this sequence; the concomitantly recorded slow LFP (i.e. 2–15 Hz); the instantaneous energy of the sLFP (calculated based on the Hilbert transform, see Methods); the fast LFP (i.e. 76–124 Hz); and the instantaneous amplitude of the fLFP. (**b**–**c**) Population data calculated from all trials tested in all recording sites of each cortical hemisphere (n = 2220 (left), n = 2530 (right)). (**b**–**c**) Show (from top to bottom): the envelope of the long distress sequence, the median post-stimulus time histograms (PSTH); the median sLFPs, the median instantaneous energy of the sLFPs, the median fLFPs; and the median instantaneous energy of the fLFPs. For calculating median population data, signals (i.e. PSTHs, LFPs, instantaneous energies) were normalized to their absolute maximum value. Grey lines indicate 25^th^ and 75^th^ percentiles. Note that the instantaneous energy of the sLFP decreased as the sequence evolved, similar to what happened to the spiking activity (see median PSTHs). On the other hand, the instantaneous energy of fLFPs followed the energy content of the sequence’s envelope. (**d**) Comparison of maximum cross-correlation coefficients calculated between the stimulus envelope, and the PSTHs and median instantaneous energy of sLFPs and fLFPs obtained in each recording site of each cortical hemisphere. Median sLFPs and fLFPs were obtained from all trials of the long distress sequence tested in each studied recording site. In the box-plots, central marks are median values, boxes indicate 25^th^ and 75^th^ percentiles, and the whiskers extend to the most extreme data points not considered outliers. The results of a parametric ANOVA are given, as well as the results of a Tukey post hoc test (***p < 0.001, Kolmogorov test for normality, p > 0.15 for all groups). Note that only the instantaneous energy of fLFPs correlated well with the envelope of the long distress sequence.

Figure 7a shows an example of the spike activity, the sLFP and the fLFP that occurred concomitantly in response to one trial of the long distress sequence. As mentioned in the preceding sections, in response to the long sequence, spiking was limited to the start of the stimulation (Fig. 7a upper panel). The sLFP recorded simultaneously to the spike activity showed a decrease in amplitude as the sequence evolved, and this decrease was also noticeable when the instantaneous energy of sLFPs was calculated as the absolute value of the Hilbert-transformed signal (Fig. 7a). A decrease in spiking and a concomitant decrease in the amplitude of sLFPs was also evident when data from single trials were pulled together for both cortical hemispheres (Fig. 7b–c). Concomitantly recorded fLFPs, on the other hand, showed no obvious decrease in amplitude as the sequence evolved. The latter was apparent in single trials (see bottom panel of Fig. 7a) and in population data from both cortical hemispheres (see bottom panel of Fig. 7b–c). The amplitude of fLFPs rather appeared to follow the fluctuations observed in the energy envelope of the long distress sequence. To summarize, it appeared as if LFP signals measured in the AC carried multiplexed information, formed by components that reacted differently to the vocal sequence, that is, low frequency LFPs followed a pattern that resembled the suppression observed in cortical spike responses, while high frequency LFPs reacted in the form of a LSSR.

We computed cross-correlations to quantify whether the fLFPs were indeed better correlated to the sequence’s envelope than were sLFPs and PSTHs. For this procedure, the median instantaneous energy of the sLFPs and fLFPs were calculated for each recording site studied (i.e. 107 in each hemisphere) by pooling data from all trials tested, and the resulting signals were cross-correlated with the sequence’s energy envelope. For calculating cross-correlations, the sequence envelope and the sLFPs and fLFPs were down-sampled to a time resolution of 1 ms, a resolution similar to that in the PSTHs. A cross-correlation was also computed between each unit’s PSTHs and the sequence’s envelope. The cross-correlation results are shown in Fig. 7d. In both cortical hemispheres, the maximum R (that is, the maximum cross-correlation coefficient obtained when temporally shifting one of the two signals being compared) was significantly higher for the fLFPs than for the sLFPs and PSTHs (repeated measures ANOVA, p < 10^−10^, Tukey post hoc test, p < 10^−10^). Both, PSTHs and median-sLFPs rendered equally low maximum R values (Tukey post hoc test, p > 0.2).

## Discussion

This manuscript characterizes spike and LFP signals recorded in the bat auditory cortex in response to distress vocal sequences. The main findings are: (i) forward suppression plays a role in the cortical processing of distress sequences, especially if the sequences are long; (ii) distress sequences evoke two concomitant phenomena: local steady-states in LFP signals, and a forward suppression that is visible in LFP and spike signals; and (iii) forward suppression and LSSRs occur in different frequency bands of cortical LFPs.

To our knowledge, this is the first report on forward suppression in response to communication sequences in the bat cortex. In a previous article, we had already shown that suppression also participates in the processing of echolocation signals^42^. In the bat AC, spiking is strongly suppressed in response to lagging segments of distress vocalization sequences, regardless of whether the sequence being heard is short or long (see Figs 2, 3, 4, 5 and Supplementary Figures S1–S3). Suppression is strong in both cortical hemispheres, with only a few neurons (i.e. less than 20%) in each hemisphere being able to respond throughout the natural distress streams. We want to stress that the cortical suppression described in this article was observed in animals that were lightly anesthetized (see Methods). Whether a similar suppression occurs in the AC of fully awake bats is something that needs to be investigated in future studies.

Besides bats, forward suppression has been described in several animal species, including rodents, birds, and monkeys^34^,^35^,^41^,^55^,^57^,^58^,^59^. When compared to other species, it appears that in the bat AC there is a higher likelihood to find “suppressed” neurons than in the rodent and bird cortex. For example, in guinea pigs, neurons that can track the temporal structure of vocal sequences are abundant in the primary and ventro-rostral belt areas of the AC^60^. Similarly, in the bird auditory system, neurons can be found that track the temporal pattern of songs, especially if what is heard is the bird’s own song^61^,^62^,^63^,^64^,^65^,^66^. It is impossible for us to establish if inter-specific differences in terms of suppression are due to different specializations for temporal processing across species, to uneven sampling of responses across cortical fields (see^57^,^60^), to differences in the stimuli used for assessing the suppression (i.e. different repetition rates, call types and sequence length), or to combinations of the aforementioned.

According to our data, there appear to be cortical asymmetries regarding the percentage of neurons that can track the distress sequences in each hemisphere. Cortical asymmetries in the auditory system have been described in a wealth of animal models^67^,^68^,^69^, including bats^70^,^71^. However, we want to stress that left-right asymmetries observed in terms of suppression in response to vocal streams need to be looked at carefully, since asymmetries are more (or only) apparent when studying responses to “simple” stimuli such as the short distress sequence used as stimulus in this article. Cortical asymmetries are less apparent when neurons are put to deal with large amounts of information, i.e. long distress sequences (see Figs 4 and 5). Note that interhemispheric differences could also potentially result from uneven sampling across cortical fields and cortical layers in each hemisphere.

The mechanisms by which cortical suppression originates were not in the focus of this study, however, based on the available literature, one could argue that suppression in response to distress sequences results from a mixture of phenomena that are known to reduce spiking at different timescales at the cortical level, such as GABA-dependent inhibition, synaptic depression, and adaptation at the thalamo-cortical synapses^32^,^33^,^34^,^35^,^36^. The finding that forward participates in the processing of distress sequences in the bat AC is not surprising from a neurobiological viewpoint, given that this feature appears to be widely distributed across vertebrate species^34^,^35^,^55^,^57^. Yet, from an ethological perspective, it is rather intriguing that cortical neurons will cease to respond to con-specific vocalizations after hearing just a few syllables. Bat distress sequences are repetitive^3^,^4^, and thus one could think that processing just the first syllables of a sequence could provide enough information for the listener to grasp the message that is being broadcasted. Future studies using non-repetitive vocal sequences need to be performed to establish if (and to what extent) forward suppression reduces spiking in response to long non-redundant vocal sequences.

Our data indicates that in response to vocal sequences, electrodes placed in the AC register a steady-state local field potential that occurs concomitantly to the forward suppression observed in spike activity. Several sources could contribute to the observed LSSR, such as the local synaptic activity, the activity generated in distant auditory areas that travels through the conductive volume until reaching the recording electrodes, and the so-called “spike-bleed through” (i.e. slow components of the spike activity that contaminate the LFPs^38^,^39^,^72^,^73^). We cannot rule out the presence of spike-bleed through in our data. However, if it occurs, spike bleed-through is more likely to contribute to steady-states measured in response to leading sequence elements, which cause the strongest response in AC neurons (see Figs 2, 3, 4, 5 and Supplementary Figures S1 and S3).

Altogether, the observed LSSR can be taken as evidence for a preserved ability to track fast time-varying sound sequences in extra-cortical (likely subcortical) structures, since LFPs measured at the level of the cortex will ultimately depend upon subcortical activity. Previous studies measuring steady-states have predicted that cortical generators play a limited role in the formation of high frequency (>40 Hz) steady-state responses, which depend more upon subcortical activity^74^. Our data supports this prediction.

LFPs measured in response to distress sequences contain multiplexed information, since they are composed by a blend of low and high frequency voltage fluctuations (see Figs 6 and 7 and Supplementary Figure S4). sLFPs and fLFPs are markedly different in their temporal dynamics, which suggests that they could be created through different sources. sLFPs could reflect local cortical processes, and, consequently, their temporal structure resembles the suppression that occurs in cortical spiking. On the other hand, fLFPs could originate from feed-forward synaptic activity and electric fields generated outside of the AC. LFPs are a popular measure of neural activity but their origin is not fully understood^39^,^40^,^75^,^76^. Knowing that certain LFP components can be better linked to local cortical processes than other components could help us to better interpret electrophysiology data, especially in the absence of concurrent measurements of LFP and spike activity.

## Methods

### Surgical procedures and recording of neuronal responses

Neuronal responses were recorded in the left and right hemispheres of 9 adult *C. perspicillata* of both sexes (3 females and 6 males). The animals used for electrophysiology experiments were taken from a breeding colony in the Institute for Cell Biology and Neuroscience, Goethe University, Frankfurt am Main (Germany). The animal use in the performed experiments was approved by the Regierungspräsidium Darmstadt (Germany, experimental permit # F104/57) and is in accordance with the Declaration of Helsinki and with current relevant guidelines and regulations for animal experimentation in Germany.

Before starting the electrophysiology experiments, bats were anesthetized subcutaneously with a mixture of ketamine (10 mg *kg ^−1^ Ketavet) and xylazine (38 mg *kg ^−1^ Rompun). A longitudinal midline incision was made through the skin overlying the skull and the underlying temporal musculature was reflected from the incision along the midline. The bat’s head was fixed using a custom-made metal rod (1 cm length, 0.1 cm diameter) that was glued onto the skull using dental cement (Paladur, Heraeus Kulzer GmbH). Neurophysiology experiments were conducted in a sound-proofed and electrically-shielded chamber. All the experiments were conducted chronically, meaning that recordings on each animal were performed for several days (maximum of 5 days). After surgery, using a scalpel blade, a hole (~3 × 2 mm) was made in the skull above the area corresponding to the auditory cortex. The position of the AC was determined from the pattern of blood vessels and landmarks in the scalp^54^. In each recording day, a bat was lightly anesthetized by injecting 1/3 of the dose used for surgery (see preceding text). Recordings started right after positioning the electrodes, but only data collected after the animal had recovered its responsivity to sound (i.e. the bats moved their outer ears in response to a loud sound produced by the researcher) were considered. Recordings lasted 4–6 h.

Neuronal responses were recorded using a wireless multichannel recording system (W16, Multi Channel Systems MCS GmbH, Germany) that recorded neuronal activity at a sampling rate of 10 kHz (per channel) and 16-bit precision. Recordings were triggered to the start of acoustic stimulation using TTL (Transistor-Transistor Logic) compatible signals produced by one of the output channels of the stimulation sound card (see below). Neuronal responses were obtained using custom-made glass electrodes, that were built by gluing together four pulled borosilicate micropipettes (GB120F-10, Science Products) so that they formed a 1 × 4 row with and inter-pipette separation (at the tips) of 500 μm (+/−50 μm). Each pipette was pulled separately using a P-97 Flaming/Brown micropipette puller (Sutter Instrument) and pipette impedances -when filled with a potassium chloride solution (3 mol*L^−1^)- were adjusted to between 5–10 MΩ. Recording electrodes were positioned in the cortex by lowering them down slowly to a distance from the cortical surface of 250–400 μm (corresponding to cortical layers III-IV). A silver wire, positioned so that it touched the dura mater of a non-auditory region of the contralateral hemisphere- was used as reference electrode. The number of neurons recorded simultaneously in single penetrations of the glass micropipette arrays spanned between one and three.

### Calculating neuronal frequency-level receptive fields

Acoustic stimuli used for probing neuronal activity were played using an Exasound E18 sound card (ExaSound Audio Design, Canada) at a sampling rate of 384 kHz (32 bit precision). The sound card was connected to an audio amplifier (Rotel power amplifier, RB-850) and signals were delivered to the bat from a calibrated speaker (ScanSpeak Revelator R2904/7000, Avisoft Bioacoustics, Berlin, Germany) located at 15 cm from the bat’s ear. The calibration curve was obtained with a ¼-inch Microphone (Brüel&Kjaer, model 4135) that was connected to a custom-made microphone amplifier.

The first step for characterizing neuronal activity was to determine the frequency-level receptive fields, by presenting pure tones of 10 ms duration (0.5 ms rise-fall time) to the contralateral ear. The frequency of the pure tones varied randomly between 10–95 kHz (5 kHz steps) and their sound pressure level (SPL) varied between a minimum of 20–50 dB SPL and a maximum of 80 dB SPL (steps of 10–20 dB SPL). SPL was online controlled based on the speaker’s calibration curve. Each of the tested frequency-level combinations was presented between 5 and 8 times.

### Distress sequences used as stimuli

Procedures used for recording distress sequences have been described in a previous article (see^4^). Two distress sequences were used to study the response of cortical neurons: a short sequence (see Fig. 1a) and a long sequence (see Fig. 1b–c). These two sequences represent typical example of distress vocalizations emitted by *C. perspicillata* (see^4^). Briefly, short distress sequences (such as the one in Fig. 1a) contain a single group of syllables that vary in their spectro-temporal design. On the other hand, long sequences (such as that in Fig. 1b–c) contain several multi-syllabic bouts, and syllables with similar spectro-temporal designs are repeated several times. The short sequence used as stimuli for this article contained seven syllables and seven syllable types (see^4^ for procedures used to define syllable types), while the long distress sequence contained 122 syllables, 8 multi-syllabic bouts, and 11 syllables types. Median syllable duration was 4.2 ms (+/−2.6) and 6.1 ms (+/−2.5) for the long and short sequences, respectively. Median repetition rate was 15.7 ms (+/−4.4) and 13.8 ms (+/−8.5) for the long and short sequences, respectively.

The two distress sequences used as stimuli were recorded from two male animals that were hand-held with their face pointing to a microphone (Brüel&Kjaer, ¼-inch Microphone 4135, Microphone Preamplifier 2670) located at 1.5 m from the bats. To encourage the production of distress calls, the researcher holding the animal softly massaged the skin behind the neck of the bats. The recording microphone used to transduce sounds into electrical impulses was powered via a custom-built microphone amplifier, and connected to a commercially available sound acquisition system (UltraSoundGate 116 Hm mobile recording interface, +Recorder Software, Avisoft Bioacoustics, Germany) for sound digitalization at 300 kHz (16 bit precision). Sequences were resampled to 384 kHz sampling rate for using them as stimuli in the electrophysiology experiments.

The short distress sequence (Fig. 1a) contained 7 syllables whose root mean square (RMS) energy, as measured at 1.5 m from the emitting bat, varied between 64–81 dB SPL (Fig. 1a, bottom panel). RMS level was calibrated to dB SPL by comparing against the RMS of a 1 kHz-94 dB SPL reference tone generated with a calibrator (Brüel & Kjaer, model 4231). The reference tone was recorded with the same microphone-amplifier-software montage used for recording the distress sequences (see preceding text). The long distress sequence used as stimuli contained 122 syllables organized into eight multi-syllabic bouts (i.e. groups of syllables, Fig. 1b). In the long sequence, the RMS level of the syllables varied between 48 and 78 dB SPL (Fig. 1b, bottom panel).

To assess the amount of energy loss between the natural distress syllables as emitted by the bats and the syllables when used as stimuli in the 20–25 kHz frequency range (the range where the peak frequency of most distress syllables occurs^4^), we applied a Tschebyscheff band-pass filter (8^th^ order) that attenuated the energy outside of the frequency range of interest. This procedure revealed an attenuation between the original distress sounds (emitted by the bats) and the same sounds when used as stimuli that amounted to a median of 6 dB and 8 dB for syllables in the short and long sequences, respectively. Based on their attenuation relative to the original recording and assuming an attenuation factor of 0.5 dB/m for frequencies close to 20 kHz^77^, one could calculate that the short and long sequences used as stimuli represent what a bat listening still at 13.5 m (short sequence) or 17.5 m (long sequence) would hear; i.e. distance-to-source = attenuation/0.5 + 1.5 m. Note that 1.5 m was the distance between the microphone and the emitting bat when recording the distress calls.

During the neurophysiology experiments, the distress sequences were played to the animals using a set-up similar to the one described in the preceding text for pure-tone stimulation. Distress sequences were segmented using custom-written Matlab scripts (MATLAB and Statistics Toolbox Release 2015b, The MathWorks, Inc., Natick, Massachusetts, United States), in a way that each segment contained only one syllable, and that the ordered concatenation of the different sequence-segments was exactly equal to the natural sequence (see segmentation points in Fig. 1a (short sequence), 1b (long sequence), and 1c (zoom-in for the second multi-syllabic bout of the long sequence)). Each sequence’s segment was saved as a *wav* file and these *wav* files were played to the animals along with the *wav* file that contained the non-segmented sequence (referred to as “natural sequence”). *Wav files* were presented in blocks with each block containing all sequence segments plus the natural sequence. In each stimulation block, *wav files* were played in the same randomized order. The interval from the end of one *wav* file to the start of the next one was fixed to 400 ms. The number of stimulation blocks spanned between 20–30 times.

To avoid acoustic artifacts while stimulating with natural sounds, the beginning and end of the *wav* files containing the sequence and the sequence-segments were multiplied by a fading window in which the energy increased linearly (at the beginning) or decreased (at the end of the *wav* files) over a time window of 0.5 ms. The absence of acoustic artifacts during the stimulation was corroborated by recording the output of the speaker, using the same microphone-software montage used for recording distress sequences from the crying bats (see preceding text). Recording the output of the speaker also allowed us to corroborate that the energy of the different sequence-segments was comparable (i.e. +/−0.1 dB) to the energy of the same segments when played in the natural sequences.

### Analysis of spike data

The data presented in this manuscript refers to spike-sorted single units. For gathering spike data, the signal recorded in each electrode was filtered between 300–3000 Hz using a 2^nd^ order Butterworth filter. Spikes were detected based on their amplitude relative to the noise level. A spike-sorting algorithm was used for grouping the spike-waveforms that, in each recording channel, appeared in response to all the tested acoustic stimulation paradigms (that is: the frequency-level receptive field, and the two tested distress sequences along with their segments). The spike-sorting procedure was based on the three principal components of the spike waveforms and the posterior application of an automatic clustering algorithm “KlustaKwik^78^,^79^. Only the spike cluster that contained the largest number of spikes was taken into consideration for further analysis.

For analysing the response to the distress sequences, the “expected responses” to the two studied sequences were constructed by re-aligning the spike-times obtained in response to the individual sequence-segments based on the position of each segment in the natural sequences. In other words, spike times were re-aligned by adding the time from the beginning of the sequence to the start of the respective segment. Spike activity in response to the sequence-segments was analysed in time windows equal to the duration of each segment + 200 ms. The time window of analysis for spike activity in response to the natural sequences was equal to the duration of the sequence + 200 ms.

Expected post-stimulus time histograms (PSTHs, 1 ms bin-size) were calculated by accumulating all the realigned spike-times observed in response to all the trials of all segments of the sequence being studied. “Observed PSTHs” were constructed from the spike times obtained in response to all trials of each natural distress sequence. Note that the “expected response” calculated here assumes that cortical neurons respond in linear manner, that is, that responses to early sequence-segments would have no influence on responses to lagging segments. To compare observed and expected responses, the observed and expected PSTHs were windowed using each sequence’s segmentation points, and the number of spikes occurring within the time borders of each segment was determined. An extra 200 ms of analysis was taken into consideration for the last segment of each sequence. The observed-to-expected, segment-dependent, spike-count ratio (abbreviated *O/E ratio*) was calculated by dividing the spike-count obtained within the time borders of each sequence segment in the observed and expected PSTHs.

### Analysis of LFP data

LFP data were analysed only for responses to the natural (non-segmented) distress sequences. Only data on LFPs obtained in response to the long sequence are reported in the main manuscript. Note that only the long sequence (of ~2 s duration) could evoke several cycles of slow oscillatory LFP activity (i.e. LFP between 2–15 Hz). Data regarding LFPs obtained in response to the short sequence can be found in Supplementary Figure S4.

To obtain LFPs, the signals recorded in each electrode were filtered between 1–200 Hz (Butterworth filter, 2^rd^ order). LFPs were offset corrected by subtracting their mean amplitude value from the amplitude value obtained at each time point of the recording. The spectral information contained in the LFPs was calculated using Welch’s periodogram method, with windows of 100 ms, 50% overlap and a frequency resolution of 4 Hz. The same analysis was used for determining the spectral content of the energy envelope of the distress sequences used as stimuli. Spectra were transformed into a log scale (i.e. in dB) and normalized to their maximum value.

We distinguished between two types of LFP signals: slow and fast LFPs (sLFPs and fLFPs, respectively). sLFPs and fLFPs were studied only in response to the long sequence, because, as mentioned in the preceding text, only the long sequence could accommodate several cycles of the slow LFP oscillations. Slow LFPs (sLFPs) were obtained by re-filtering LFP data between 2–15 Hz (corresponding to the theta and alpha bands of the electroencephalogram). Fast LFPs (fLFPs) were calculated by filtering LFPs between 76–124 Hz, which corresponds to the best represented range of high frequencies (>50 Hz) in the energy envelope of the long sequence (see Fig. 6f in the results section). The instantaneous amplitude of sLFPs and fLFPs was calculated as the absolute value of the Hilbert-Transform of LFPs obtained in individual trials.

## Additional Information

**How to cite this article**: Hechavarría, J. C. *et al*. Vocal sequences suppress spiking in the bat auditory cortex while evoking concomitant steady-state local field potentials. *Sci. Rep.* **6**, 39226; doi: 10.1038/srep39226 (2016).

**Publisher's note:** Springer Nature remains neutral with regard to jurisdictional claims in published maps and institutional affiliations.

## Supplementary Material

Supplementary Figures
