# Supplementary material for: Vocal sequences suppress spiking in the bat auditory cortex while evoking concomitant steady-state local field potentials
Source: Sci Rep. 2016 Dec 15;6:39226. doi: 10.1038/srep39226 (PMC5156950; doi:10.1038/srep39226)
Supplement: Supplementary Figures [file srep39226-s1.pdf]

***Title*: Vocal sequences suppress spiking in the bat auditory cortex while evoking concomitant steady-state local field potentials**

Authors: Julio C. Hechavarría (*) [1], M. Jerome Beetz[1], Silvio Macias[1,2], Manfred Kössl [1].

Affiliations: [1:] Institut für Zellbiologie und Neurowissenschaft, Goethe-Universität, Frankfurt/M., Germany. [2:] Department of Psychological & Brain Sciences, Johns Hopkins University, Baltimore, USA.

* Corresponding author

***Mailing address*:**

Julio C. Hechavarría

Email: Hechavarria@bio.uni-frankfurt.de

Institut für Zellbiologie und Neurowissenschaft, Max-von-Laue-Straße 13, 60438,

Frankfurt/Main, Germany, Tel.: +49 69798 42066

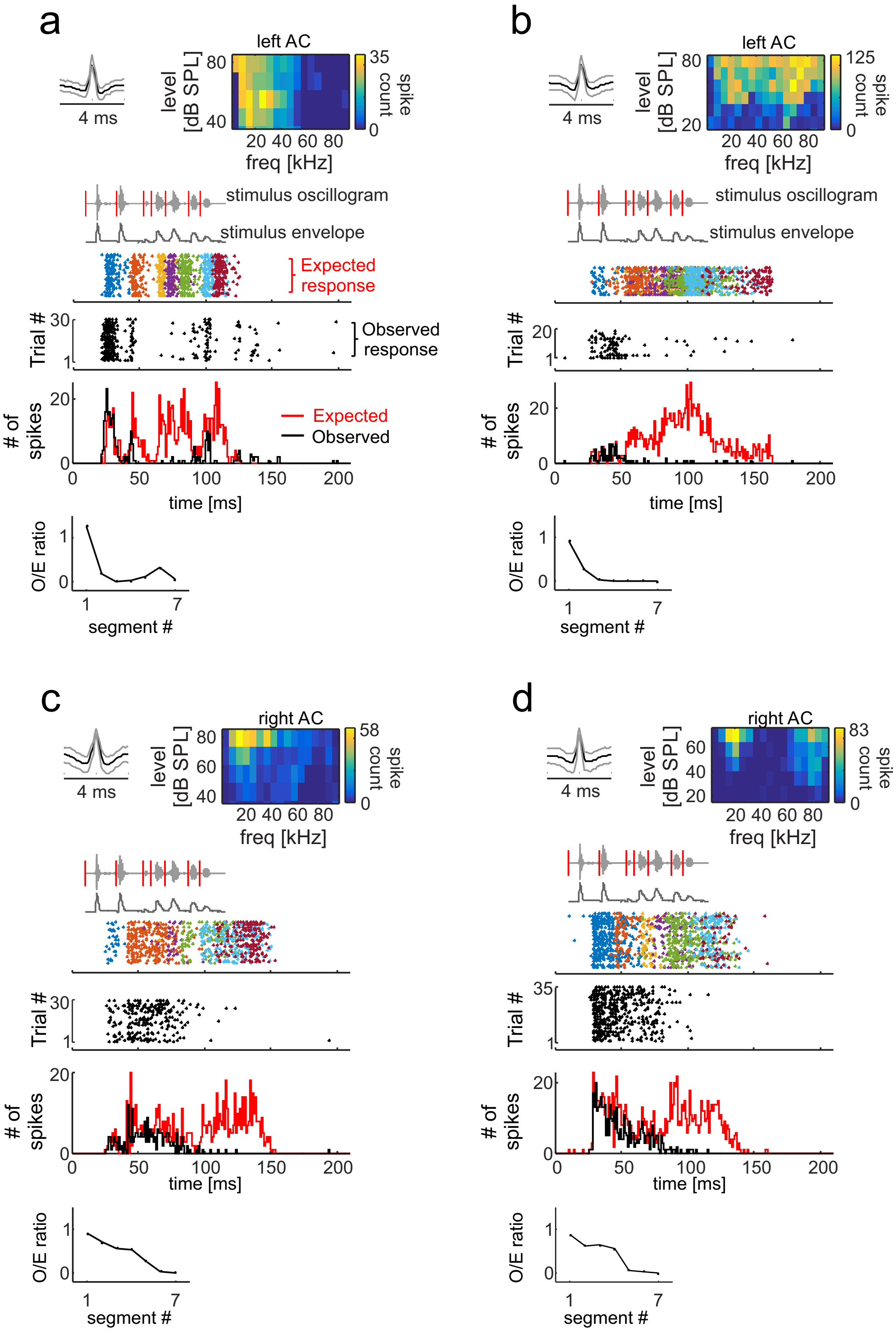

a
b
c
d
left AC
right AC
level [dB SPL]
freq [kHz]
spike count
4 ms
stimulus oscillogram
stimulus envelope
Expected response
Observed response
Trial #
# of spikes
Expected
Observed
time [ms]
O/E ratio
segment #

Figure S1. Four example neruons (**a, b, c, d**) that showed suppression in response to a short distress sequence. All four subpanels are organized in a similar way, and they show (from top to bottom): the median spike waveform (25th and 75th percentiles represented in grey) and the frequency-level receptive field of each neuron; the oscillogram and energy envelope of the sequence used as stimulus; the raster plot of the expected response (coloured dots); the raster plot of the observed response (black dots); the post-stimulus time histograms calculated for the expected (red) and observed responses (black); and the segment-dependent *observed-to-expected* spike-count ratio (*O/E ratio*).

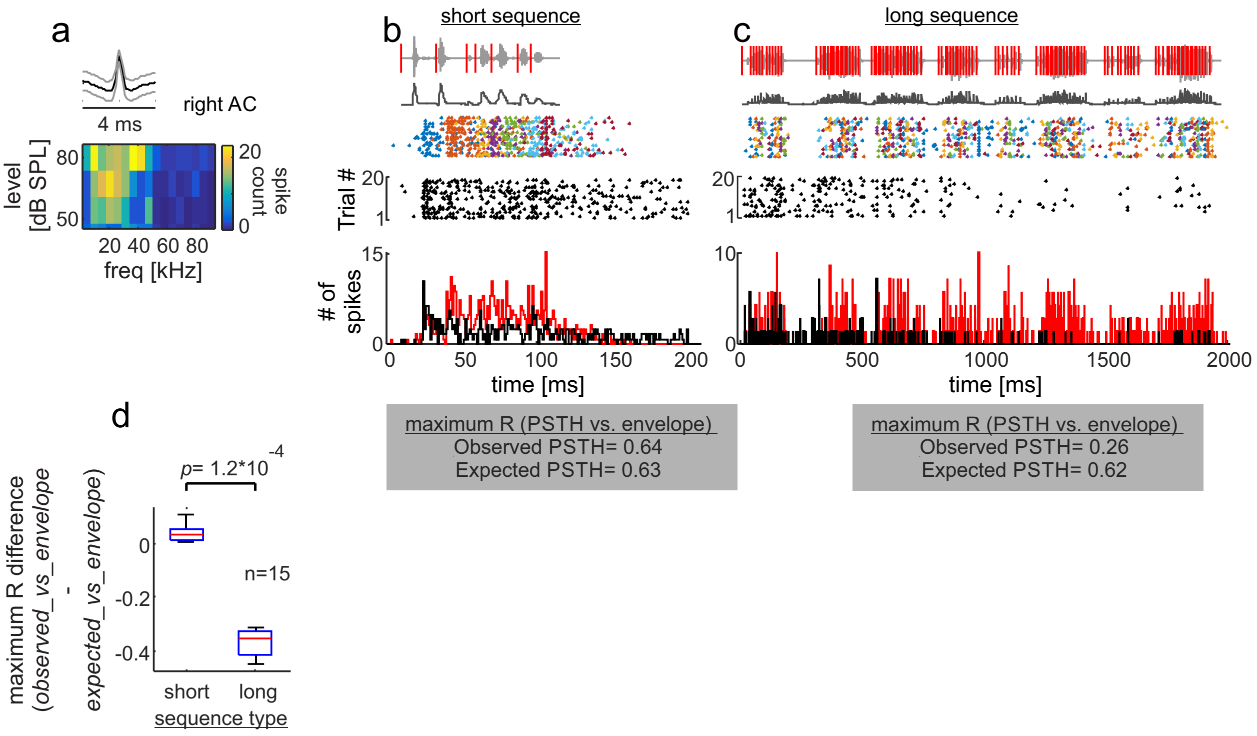

Figure S2. An example neuron that was capable of firing throughout the short distress sequence, but that showed strong suppression in response to the long sequence. (**a**) Shows the frequency-level receptive field and spike shape of the neuron. (**b**) Shows the response to the neuron to the short sequence. (**c**) Shows the response to the long sequence. In **b** and **c**, the top, middle, and bottom panels show: the stimulus oscillogram and envelope and the raster plot of the expected response (top); the raster plot of the observed response (middle), and the post-stimulus time histograms (PSTHs) of the expected and observed responses (bottom). (**d**) Shows a comparison of the difference in maximum cross-correlation coefficient (maximum R) obtained between the envelope of each stimulus sequence and the observed and expected PSTHs obtained with the sequence in question. The *p* value refers to a paired *signrank* test. Differences in maximum R were studied only in 15 neurons in which both sequences were studied, and that behaved as

“followers” in response to the short sequence (i.e. their expected and observed PSTHs correlated equally well with the short sequence’s envelope).

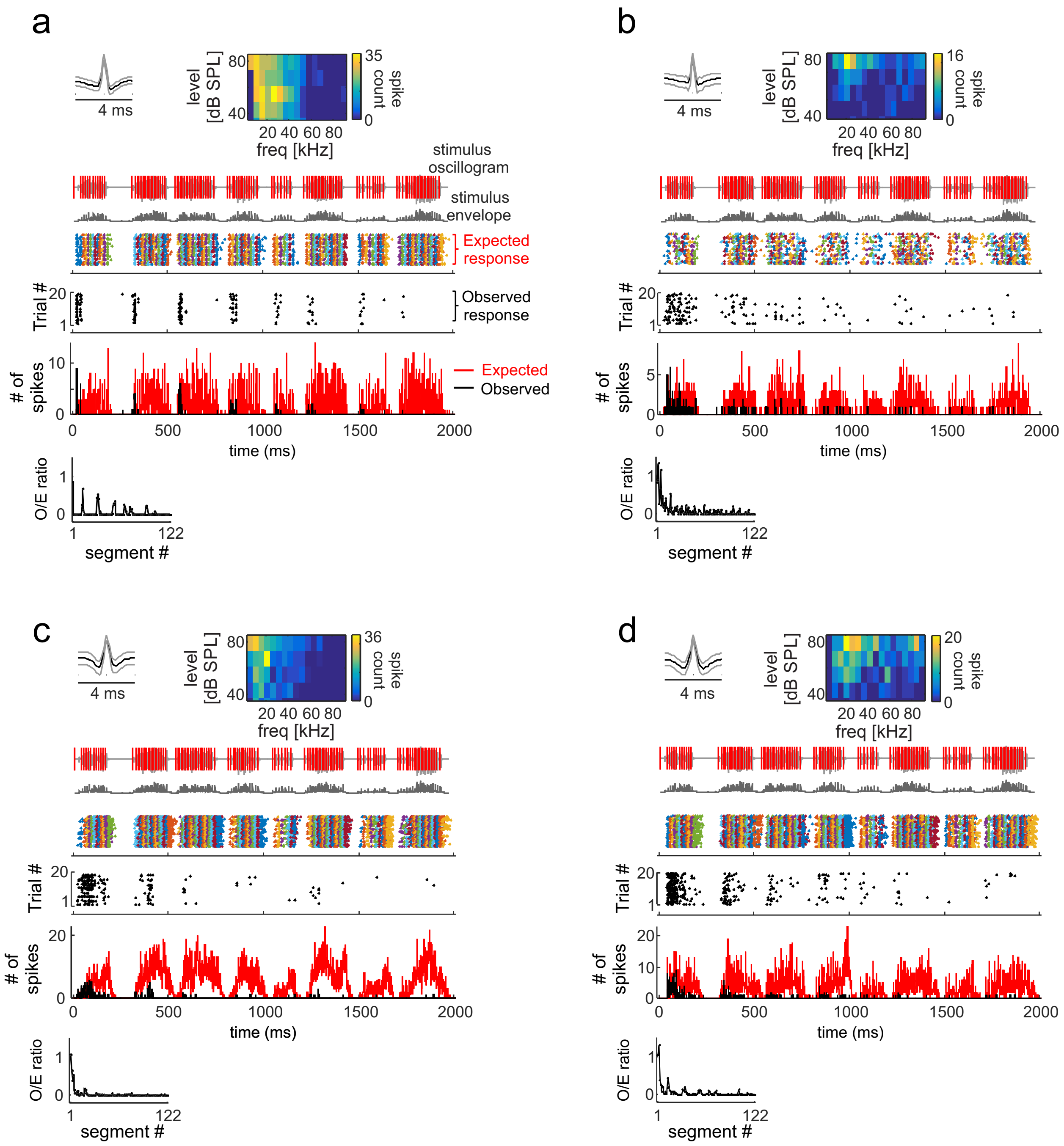

Figure S3. Four example neruons (**a, b, c, d**) that showed suppression in response to the long distress sequence. All four subpanels are organized in a similar way, and they show (from top to bottom): the median spike waveform (25th and 75th percentiles represented in grey) and the ferquency-level receptive field of each neuron; the oscillogram and energy envelope of the long sequence used as stimulus; the raster plot of the expected response (coloured dots); the raster plot of the observed response (black dots); the post-stimulus time histograms (PSTHs) calaculated for

the expected (red) and observed responses (black); and the segment-dependent observed-to-expected spike-count ratio (*O/E ratio*). Note that the neuron reprsented in **a** is the same neuron whose response is represented Fig. S1a in response to the short sequence.

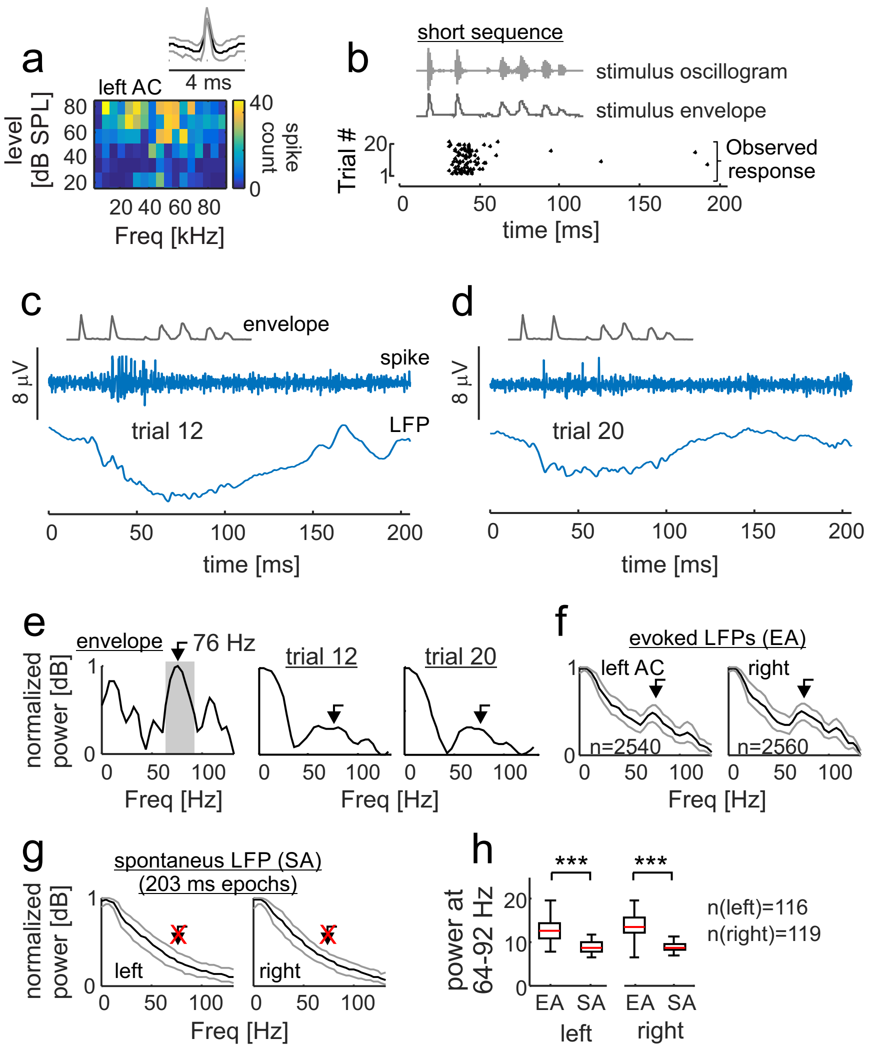

Figure S4. Steady-state local field potentials (LFPs) in response to a short distress sequence. **(a)** Spike waveform (median in black, 25th and 75th percentiles in grey) and frequency-level receptive field of one unit from the left auditory cortex. **(b)** From top to bottom: the oscillogram and energy envelope of the short sequence, and the raster plot of the response obtained when playing the sequence ("observed response") for the same neuron represented in **a**. **(c-d)** Show spike and LFP waves obtained in the same neuron represented in **a-b** during two trials of the short sequence. **(c-d**) Show (from top to bottom): the energy envelope of the short sequence; the spike waves (i.e. neural signals filtered between 300-3000 Hz), and the concomitant LFP (i.e. signals filtered between 1-200 Hz). **(e)** Shows the spectrum calculated from the energy envelope of the short sequence, and the spectrum of the LFPs recorded in the two trials represented in **c-d**.

Spectra are represented in decibels and normalized to their maximum value. Note that there was a peak in the envelope's spectrum at ~ 76 Hz, and a similar peak was found in spectra from individual trials. **(f)** The median normalized spectrum obtained from all trials studied in each cortical hemisphere. Grey lines represent 25th and 75th percentiles. **(g)** The median spectra of LFP epochs recorded in the absence of acoustic stimulation (spontaneous activity, SA). The duration of SA epochs was similar to that used for studying epochs of evoked activity (EA). There was no 76 Hz peak in the average SA spectra. (**h**) Comparison of median LFP power obtained in the 64-92 Hz LFP band, the band that enclosed the strongest energy fluctuations in the short sequence's envelope (see shaded area in **e**). Median power was calculated from all the trials studied in a given recording site, with and without acoustic stimulation (EA and SA, respectively). In the box-plots, central marks are medians, boxes indicate 25th and 75th percentiles and whiskers extend to the most extreme data points not considered outliers. The results of paired Wilcoxon Signed Rank tests are given (***= $p<0.001$).
